# Genome Sequences of Rhodoplanes serenus and Two Thermotolerant Strains, Rhodoplanes tepidamans and “Rhodoplanes cryptolactis,” Further Refine the Genus

**DOI:** 10.1128/mra.00099-23

**Published:** 2023-06-05

**Authors:** Amiera A. Rayyan, John A. Kyndt

**Affiliations:** a College of Science and Technology, Bellevue University, Bellevue, Nebraska, USA; University of Delaware College of Engineering

## Abstract

*Rhodoplanes* is a small genus of anoxygenic purple nonsulfur bacteria belonging to the family *Nitrobacteraceae* in the class *Alphaproteobacteria*. We sequenced the genomes of one mesophilic and two thermotolerant strains, and whole-genome-based comparisons confirmed suspected close similarities and synonyms between different species in the genus.

## ANNOUNCEMENT

The phototrophic genus *Rhodoplanes* contains 10 named species (1); however, until now, only 3 had been sequenced: Rhodoplanes piscinae, R. roseus, and R. elegans (2). We have now sequenced the genomes of Rhodoplanes serenus, R. tepidamans, and “*Rhodoplanes cryptolactis*.” Earlier studies proposed that the species *R. cryptolactis* be included in the new species *R. tepidamans*, based on the loss of *R. cryptolactis* from the ATCC collection (3). Whole-genome sequencing was performed to determine the genomic differences between the known strains of *Rhodoplanes* and to further refine the genus.

Rhodoplanes serenus DSM 18633 was originally isolated from pond water at the University of Tokyo, Japan (4), while *R. tepidamans* DSM 9987 and *R. cryptolactis* are thermotolerant species isolated from a soil sample near a hot spring in Wyoming (5). Genomic DNA (gDNA) of *R. serenus* and *R. tepidamans* was obtained from the DSMZ culture collection, while frozen cultures of *R. cryptolactis* TEM were obtained from T. E. Meyer, who received a direct transfer from the original isolation by R. Stadtwald-Demchick, F. R. Turner, and H. Gest (5). The cultures were grown in DSMZ medium 27, supplemented with 20 μg/L vitamin B21, and DNA was extracted using the GeneJET genomic DNA isolation kit (Thermo Scientific). DNA analysis showed ratios of absorption at 260/280 nm of 1.96 for *R. serenus*, 1.85 for *R. tepidamans*, and 2.0 for *R. cryptolactis*.

Sequencing libraries were prepared using the Illumina Nextera DNA Flex library prep kit and sequenced using an Illumina MiniSeq instrument, using 500 μL of a 1.8 pM library. Paired-end (2 × 150-bp) sequencing generated 3,015,458 reads and 227 Mbp for *R. serenus*, 1,239,460 reads and 187.2 Mbp for *R. tepidamans*, and 3,081,022 reads and 465.2 Mbp for *R. cryptolactis*. Quality control of the reads was performed using FastQC version 1.0.0 within BaseSpace (Illumina), using a k-mer size of 5 and contamination filtering. We assembled the genome *de novo* through BV-BRC (6) using Unicycler (7). A comparison of the features of these new genomes to the other *Rhodoplanes* genomes is provided in Table 1. The genomes were annotated using the NCBI Prokaryotic Genome Annotation Pipeline (8). The resulting coding sequences and tRNAs are also available in Table 1. Default parameters were used for all software applications unless otherwise noted.

**TABLE 1:** Overview of the features of all the *Rhodoplanes* genome sequences

Species	Genome size (bp)	GC content (%)	No. of contigs^*a*^	*N*_50_ (bp)	Coverage (×)	No. of CDSs^*b*^	No. of tRNAs	Reference	GenBank accession no.
*R. tepidamans* DSM 9987	6,800,548	71.1	132	94,445	28	6,095	48	This study	JAQQLI000000000
*R. cryptolactis* TEM	6,781,861	71	238	50,670	69	6,150	48	This study	JAQQSA000000000
*R. roseus* DSM 5909	6,879,536	69.4	1,040	19,390	308	6,585	48	9	NPEX00000000
*R. elegans* DSM 11907	6,538,588	69.5	320	32,496	33	6,093	48	2	NHSK00000000
*R. serenus* DSM 18633	5,501,108	70.3	51	262,047	41	4,965	49	This study	WNKV00000000
*R. piscinae* DSM 19946	5,370,996	70.2	880	15,387	385	5,201	46	10	NPEW00000000

a Number of contigs >300 bp.

b CDSs, coding DNA sequences.

**FIG 1:**
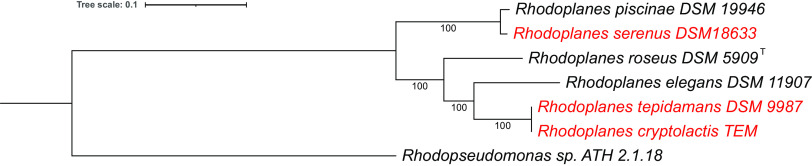
Whole-genome-based phylogenetic tree of all sequenced *Rhodoplanes* species. The phylogenetic tree was generated using the codon tree method within BV-BRC (6), which used PGfams as homology groups; 875 PGfams were found among these selected genomes using the CodonTree analysis, and the aligned proteins and coding DNA from single-copy genes were used for RAxML analysis (12, 13). One hundred rounds of the rapid bootstrapping option in RAxML were used to generate the support values for the phylogenetic tree. The branch length tree scale is defined as the mean number of substitutions per site, which is an average across both nucleotide and amino acid changes. New genomes are in red. The *Rhodopseudomonas* sp. strain ATH 2.1.18 genome was used as an outgroup (17). iTOL was used for the tree visualization (18).

A JSpecies comparison (11) of the average nucleotide identity (ANIb) showed that the 3 new genomes all have ≤86% ANI with the earlier sequenced *Rhodoplanes* genomes, except for *R. serenus* and *R. piscinae*, which share 97.4% ANI. The latter is above the proposed 95% cutoff for genome definition of a species (11). *R. tepidamans* and *R. cryptolactis* are about equidistant to all the *Rhodoplanes* species; however, they share 99.9% ANI, which indicates that they belong to the same species. Whole-genome-based phylogenetic analysis was performed using RAxML (12, 13) version 8.2.11 with all the *Rhodoplanes* genomes (Fig. 1). Protein sequences were aligned using MUSCLE (14), and the nucleotide coding gene sequences were aligned using the Codon_align function of Biopython (15). A concatenated alignment of all proteins and nucleotides was written to a partition file for RAxML. Consistent with the ANI analysis, this analysis also closely grouped *R. serenus* with *R. piscinae* and *R. tepidamans* with *R. cryptolactis*. This finding agrees with the suggestion that *R. piscinae* should be a later synonym of *R. serenus* (16) and confirms that *R. tepidamans* and *R. cryptolactis* belong to the same species. The addition of these new *Rhodoplanes* genomes has substantially strengthened the phylogenetic tree of this genus.

### Data availability.

These whole-genome shotgun projects have been deposited at DDBJ/ENA/GenBank under the accession numbers WNKV00000000 for *R. serenus*, JAQQLI000000000 for *R. tepidamans*, and JAQQSA000000000 for *R. cryptolactis*. The versions described in this paper are versions WNKV01000000, JAQQLI010000000, and JAQQSA010000000, respectively. The raw sequencing reads have been submitted to the SRA under the accession numbers SRR23308057 for *R. serenus*, SRR23308056 for *R. tepidamans*, and SRR23308055 for *R. cryptolactis*.
